# Official incidence and trends of occupational musculoskeletal disorders in Turkey in 2014–2024: implications of possible underreporting

**DOI:** 10.2478/aiht-2026-77-4133

**Published:** 2026-09-25

**Authors:** Elif Reyhan Şahin, Yağmur Kınacı Gümüşçubuk

**Affiliations:** Erzurum City Hospital, Occupational Medicine Clinic, Erzurum, Turkey; Hacettepe University Medical Faculty, Department of Public Health, Division of Occupational Medicine, Ankara, Turkey

**Keywords:** chronic crepitant synovitis, epidemiology, gender-specific patterns, ICD-10, lateral epicondylitis, occupational disease burden, public health surveillance, SGK, epidemiologija, epidemiološko praćenje javnog zdravstva, kronični krepitirajući sinovitis, lateralni epikondilitis, MKB-10, opterećenje profesionalnim bolestima, rodni trendovi, SGK

## Abstract

In Turkey, official incidence figures for work-related musculoskeletal disorders (MSDs) remain notably low despite established ergonomic risks. The aim of this longitudinal study was to determine national incidences of all recognised occupational MSD cases among insured workers included in the publicly available registry of the Social Security Institution of Turkey (SGK) between 2014 and 2024, to calculate annual rates per million insured workers, and to establish temporal, gender-specific, and diagnostic trends. A total of 744 cases were recorded, with recognised incidence increasing from 1.74 per million in 2014 to a peak of 9.03 in 2021. Lateral epicondylitis and chronic crepitant synovitis were the most frequent diagnoses. Whilst male workers accounted for most cases, female incidence rates equalled or exceeded male rates in 2020–2021, coinciding with the COVID-19 pandemic outbreak. Our findings point to structural flaws and barriers in the current, compensation-based registry system that may result in under-recognition and consequent underreporting of occupational MSDs, which calls for strengthening prevention-oriented surveillance to inform robust healthcare policies.

## Abstract (hr)

U Turskoj je službena incidencija profesionalnih mišićno-koštanih poremećaja (MKP) i dalje izrazito niska, unatoč dobro poznatim ergonomskim rizičnim čimbenicima. Cilj ovog longitudinalnog istraživanja bio je utvrditi nacionalnu incidenciju svih priznatih slučajeva profesionalnih mišićno-koštanih poremećaja među osiguranim radnicima, evidentiranima u javno dostupnom registru Turskoga zavoda za socijalno osiguranje (SGK) od 2014. do 2024. godine kako bi se izračunale godišnje stope na milijun osiguranih radnika te analizirali vremenski, rodni i dijagnostički trendovi. Ukupno su zabilježena 744 slučaja, pri čemu se priznata incidencija povećala s 1,74 na milijun osiguranih radnika u 2014. godini do najveće vrijednosti od 9,03 u 2021. godini. Najčešće dijagnoze bile su lateralni epikondilitis i kronični krepitirajući sinovitis. Iako je većina slučajeva zabilježena među muškarcima, stope incidencije u žena bile su jednake ili više od stopa u muškaraca tijekom 2020. i 2021., što se podudara s izbijanjem pandemije bolesti COVID-19. Dobiveni rezultati upućuju na strukturne nedostatke i prepreke postojećeg sustava registracije koji se temelji na priznavanju prava na naknadu, što može dovesti do nedovoljnog prepoznavanja, a posljedično i potprijavljivanja profesionalnih mišićno-koštanih poremećaja. To upućuje na potrebu jačanja preventivno usmjerenog epidemiološkog praćenja radi oblikovanja učinkovitijih zdravstvenih politika.

According to the Joint Estimates of the Work-related Burden of Disease and Injury for 2000–2016 issued by the World Health Organization (WHO) and International Labour Organization (ILO) (1), work-related risk factors were responsible for an estimated 1.9 million deaths and nearly 90 million disability-adjusted life years (DALYs) globally. These figures underscore that occupational diseases constitute a substantial component of the global health burden attributable to workplace exposures. Among these outcomes, musculoskeletal disorders (MSDs) – driven by ergonomic stressors such as repetitive movements and cumulative biomechanical stress – seem to be among the most prevalent (2). Furthermore, epidemiological evidence consistently demonstrates the aetiologic importance of ergonomic stressors (3), and the global burden of MSDs is a leading contributor to years lived with disability (4,5,6).

In Turkey, however, nationwide data from the Social Security Institution (Sosyal Güvenlik Kurumu; SGK) (7) yield low incidence rates compared to high prevalence rates of musculoskeletal symptoms reported by field studies in the manufacturing and healthcare sectors (8,9,10). Epidemiologically, these differences reflect fundamentally different dimensions of the disease burden; a low incidence rate of newly certified cases can naturally coexist with a high prevalence of long-lasting chronic conditions in the active workforce, but this substantial discrepancy extends beyond the chronic nature of MSDs alone and may point to underreporting owed to organisational or policy flaws and stringent institutional barriers to formal recognition of occupational MSD cases.

Currently, there is a lack of long-term analysis that would evaluate the trends of official figures over the last decade. This would be particularly relevant for identifying the effects of major shifts in national occupational health regulation (11) and of changing industrial demands. By 2014, regulatory updates have introduced standardised reporting protocols across all employment sectors and established an integrated electronic network directly linking diagnostic healthcare facilities to the central SGK database.

The aim of our study was to analyse national incidence trends of officially recognised occupational MSD cases in Turkey between 2014 and 2024 and to identify temporal patterns, point to potential systemic surveillance gaps, and provide arguments for a shift from a compensation-based model towards a prevention-oriented occupational health policy.

## METHODS

We utilised publicly accessible SGK data retrieved from the official statistical yearbooks for years 2014–2024. The SGK registry is a mandated anonymised database based on the national Regulation on the Procedures and Principles for Determining Loss of Working Capacity and Earning Capacity in the Profession (11). Under this regulatory framework, occupational MSDs are categorised as “Group E: Occupational Diseases Caused by Physical Factors”, each coded after the 10^th^ revision of the International Statistical Classification of Diseases and Related Health Problems (ICD-10). However, there is also a “Group Z: Another Disease Not on the List”, which does not match strict definitions. “Group Z” operates as an ‘open system’ that allows the SGK Social Insurance Health Board and the Higher Health Board to evaluate unlisted conditions on a case-by-case basis using clinical and workplace evidence. Workers certified under Group Z receive the exact same legal recognition, disability benefits, and financial compensation as those with standard list diagnoses. Since the SGK database does not specify diseases for this group, we could not identify possible MSD cases that may have ended there. We shall address this issue later in discussion.

### Study population and inclusion criteria

The study population included all workers insured under the social and universal health insurance law (No. 5510, Article 4/1-a) (12) between 1 January 2014 and 31 December 2024 who underwent full legal medical evaluation and whose MSD was officially recognised as "occupational" by an appointed SGK health committee. The study population did not, however, include any workers beyond these, such as self-employed or unregistered workers.

### Case definition and disease classification

The analysis included all the ICD-10 diagnoses recognised by the SGK as work-related MSDs between 2014 and 2024 (Table 1). All recorded diagnoses fell exclusively within soft tissue disorders (M60–M79), and no other occupational MSD diagnoses were recorded over that period, such as dorsopathies (M40–M54) or arthropathies (M15–M19).

**Table 1:** ICD-10 classification of musculoskeletal disorders recorded as occupational by the Turkish Social Security Institution (SGK) between 2014 and 2024

**ICD-10 code**	**Diagnosis**
M65.04	Tendon sheath abscess
M70.0	Chronic crepitant synovitis
M70.2	Olecranon bursitis
M70.4	Prepatellar bursitis
M77.0	Medial epicondylitis
M77.1	Lateral epicondylitis

ICD-10 – International Statistical Classification of Diseases and Related Health Problems, 10^th^ revision. All officially recorded cases during this period fell exclusively within the M60–M79 category (soft tissue disorders)

According to the administrative protocols of the SGK registry, if an individual worker is diagnosed with multiple concurrent occupational MSDs (e.g. simultaneous bilateral carpal tunnel syndrome and epicondylitis) during the same medical board evaluation, these conditions are consolidated under a single case file for that calendar year. Consequently, each affected worker is quantified as a single case, ensuring that the reported incidence rates reflect person-based counts and are not inflated by multiple concurrent diagnoses for the same individual.

### Data processing and outcome measures

The primary outcome was the annual registry-based incidence rate of occupational MSDs, expressed per million of insured workers. The secondary outcome was the proportion of MSDs relative to all occupational diseases recognised by the SGK. All temporal trends were evaluated based on standard calendar years, and population metrics were defined strictly as the annual counts of active insured workers registered within the national database. Annual incidence rates were calculated using the following equation:

[1] $$\text{Incidence}\;\text{rate}=\left(\frac{\text{number}\;\text{of}\;\text{occupational}\;\text{MSD}\;\text{cases}\;\text{in}\;\text{a}\;\text{given}\;\text{year}}{\text{number}\;\text{of}\;\text{insured}\;\text{workers}\;\text{in}\;\text{the}\;\text{same}\;\text{year}}\right)\times 1,000,000$$

This calculation ensured consistency with the population at risk as defined by SGK.

### Statistical analysis

The data were compiled and analysed using Microsoft Excel, version 2021 (Microsoft Corp., Redmond, WA, USA). Descriptive statistics were utilised to summarise case counts, registry-based incidence rates, and proportional distributions.

To evaluate and quantify the temporal trends over the 11-year period (2014–2024), we relied on linear regression analysis, treating the calendar years as the independent variable and the annual registry-based incidence rates as the dependent variable. The slope of the regression line was used to assess the direction and magnitude of the trend, and a p-value of <0.05 was considered statistically significant.

To ensure the reliability of the findings, we ran independent calculations of all rates and internal consistency checks between the raw yearbook data and the final dataset.

### Ethical considerations

As this research involved the analysis of publicly available, aggregated, and anonymised administrative data, it did not involve direct intervention with human participants and therefore did not require ethical approval or informed consent.

## RESULTS

### Overall and gender-specific trends

**Figure 1:**
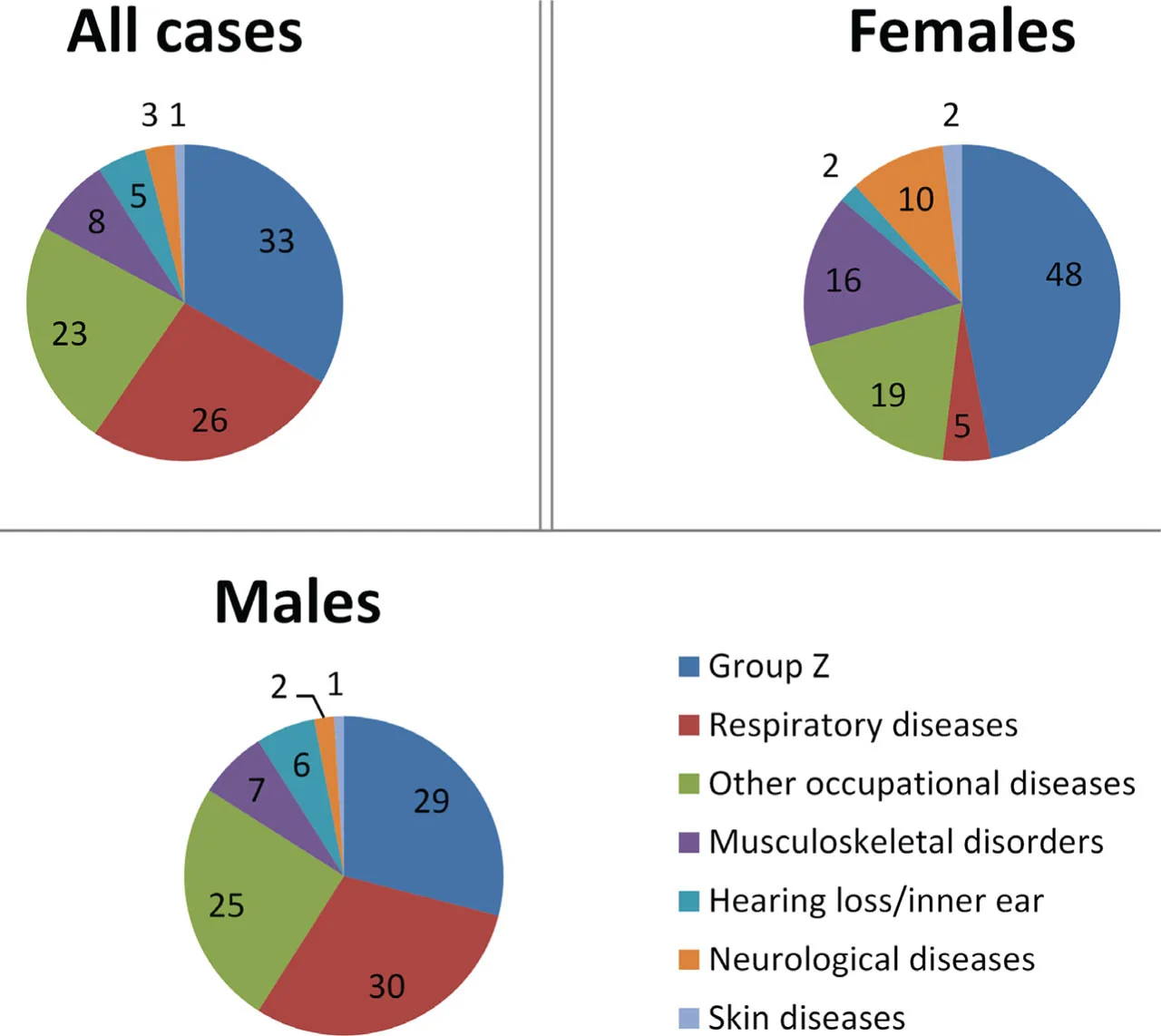
Distribution of recognised occupational disease categories in Turkey over 2014–2024 [based on author-generated analyses of SGK registry data]. “Group Z (list-outside)” denotes cases legally certified as occupational diseases through institutional adjudication, even though they are not explicitly listed in standard regulatory diagnostic categories. “Other occupational diseases” includes certified cases belonging to defined statutory subcategories within the standard regulatory list (Groups A–E) that fall outside the major organ-system categ ories shown (respirator y, neurological, musculoskeletal, hearing, or skin disorders)

Between 2014 and 2024, the SGK registry recorded a total of 744 recognised occupational MSD cases, which accounted for 8.0 % of all occupational diseases in the entire population and showed a clear gender disparity. They accounted for 7.0 % of all occupational diseases among men and for as much as 16.0 % of all occupational diseases among women (Figure 1). The “Group Z (*Z Grubu*, in Turkish)” designates all other diseases not on the standard diagnostic list, which are recognised by medical boards separately upon appeal.

**Figure 2:**
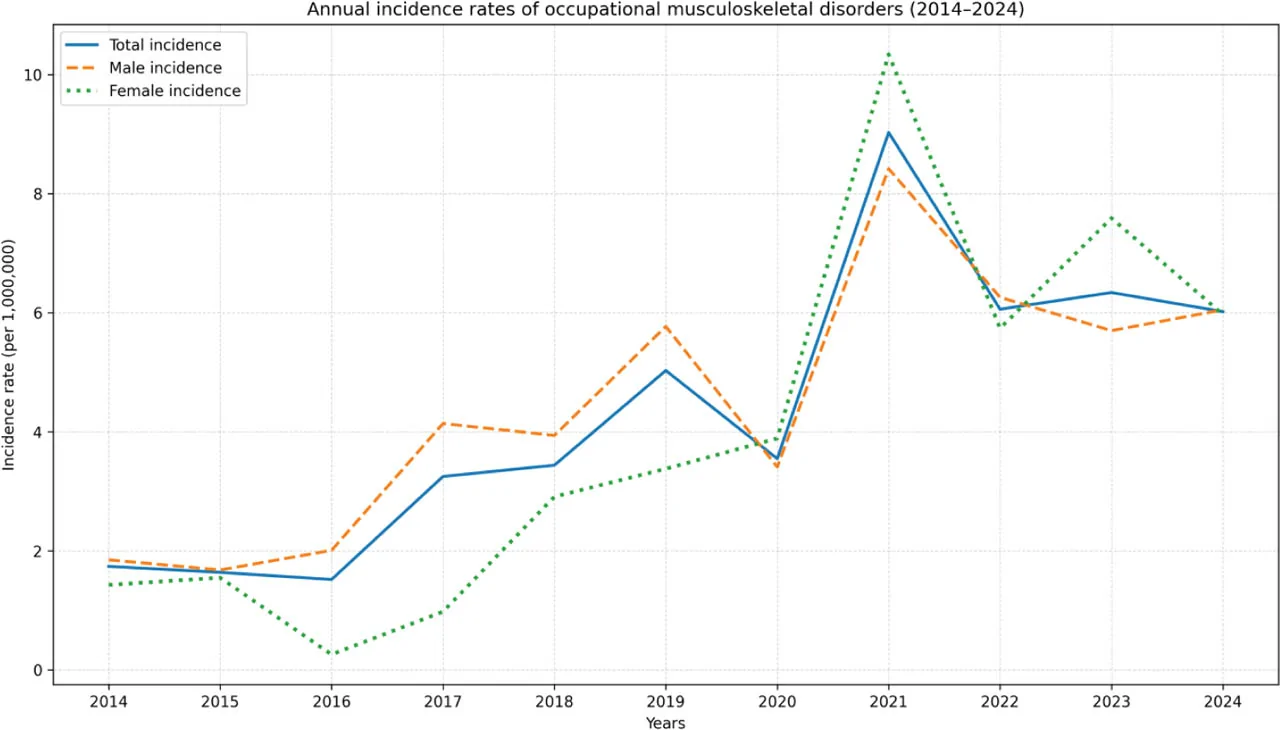
Trends in the incidence of officially recognised occupational musculoskeletal disorders in Turkey between 2014 and 2024

Table 2 shows the annual incidence of occupational MSD cases among insured workers, totalling 744 recognised cases, of which 522 (70.2 %) occurred among male and 222 (29.8 %) among female workers. The overall incidence rates show a clear upward trend, increasing significantly from 1.74 per million in 2014 to 9.03 per million in 2021 (R^2^=0.686, β=0.595, p=0.002). Following the 2021 peak, they declined but remained substantially higher than early-period levels, stabilising between 6.02 and 6.34 per million from 2022 to 2024 (Table 2, Figure 2).

**Table 2:** Number of insured workers and the incidence of officially recognised occupational musculoskeletal disorder cases in Turkey between 2014 and 2024

**Year**	**Insured workers (total)**	**Insured workers (male)**	**Insured workers (female)**	**Occupational MSD cases (total)**	**Occupational MSD cases (male)**	**Occupational MSD cases (female)**	**Incidence (per million)**
2014	13,240,122	9,742,995	3,497,127	23	17	6	1.74
2015	13,999,398	10,133,702	3,865,696	23	16	7	1.64
2016	13,775,188	9,949,970	3,825,218	21	20	1	1.52
2017	14,477,817	10,389,595	4,088,222	47	43	4	3.25
2018	14,229,170	9,895,701	4,333,469	49	39	10	3.44
2019	14,314,313	9,875,479	4,438,834	72	57	15	5.03
2020	15,203,423	10,574,303	4,629,120	54	36	18	3.55
2021	16,169,679	11,046,133	5,123,546	146	93	53	9.03
2022	17,332,991	11,656,553	5,676,438	105	73	32	6.06
2023	16,406,420	10,871,438	5,534,982	104	62	42	6.34
2024	16,602,868	10,910,087	5,692,781	100	66	34	6.02

Between 2014 and 2019, the incidence rates were consistently higher in male than female workers (Figure 2). However, the COVID-19 pandemic brought a notable shift, and in 2021, female worker incidence rate peaked at 10.34 per million, slightly exceeding the male rate of 8.42 per million for the first time. From 2022 onward, male incidence returned to its historically higher rates and continued to be higher through 2024, while female rates declined to 5.97 per million.

Table 3 shows high heterogeneity in the distribution of occupational MSD diagnoses by year and gender. Lateral epicondylitis (ICD-10: M77.1) was the most frequent diagnosis (266 cases over 11 years), with annual incidence soaring from 10 in 2014 to 49 in 2021, followed by chronic crepitant synovitis (M70.0) (255 cases) which peaked at 56 cases in the same year. Medial epicondylitis (M77.0) and tendon sheath abscess (M65.04) showed a gradual upward trend, whereas other diagnoses remained relatively uncommon but consistently reported throughout the study period.

**Table 3:** Distribution of occupational musculoskeletal disorder diagnoses recognised by the Turkish administrative compensation registry between 2014 and 2024

**Diagnosis (ICD-10)**	**Gender**	**2014**	**2015**	**2016**	**2017**	**2018**	**2019**	**2020**	**2021**	**2022**	**2023**	**2024**
Tendon sheath abscess (M65.04)	Male	1	1	2	4	1	9	5	10	8	7	7
	Female	1	1	0	1	2	2	2	2	2	4	5
	Total	2	2	2	5	3	11	7	12	10	11	12
Chronic crepitant synovitis (M70.0)	Male	7	5	9	11	7	14	6	30	24	21	17
	Female	2	4	0	0	3	10	7	26	14	25	13
	Total	9	9	9	11	10	24	13	56	38	46	30
Olecranon bursitis (M70.2)	Male	0	0	0	1	2	6	2	3	4	3	3
	Female	0	0	0	0	0	0	1	2	0	1	2
	Total	0	0	0	1	2	6	3	5	4	4	5
Prepatellar bursitis (M70.4)	Male	1	0	0	2	4	1	1	5	3	1	2
	Female	0	0	0	0	0	0	1	4	0	1	1
	Total	1	0	0	2	4	1	2	9	3	2	3
Medial epicondylitis (M77.0)	Male	0	1	0	8	5	4	4	11	10	8	13
	Female	1	1	0	1	1	0	0	4	7	6	4
	Total	1	2	0	9	6	4	4	15	17	14	17
Lateral epicondylitis (M77.1)	Male	8	9	9	17	20	23	18	34	24	22	24
	Female	2	1	1	2	4	3	7	15	9	5	9
	Total	10	10	10	19	24	26	25	49	33	27	33

* ICD-10, International Classification of Diseases, 10^th^ revision

## DISCUSSION

Our analysis of the official SGK registry data reveals consistently low incidence rates of recognised occupational MSDs in Turkey over the 2014–2024 period, which is lower than we expected, considering the ergonomic risks associated with certain industrial sectors (13, 14), nationwide estimates (15, 16), and global occupational exposure data (17, 18). A similar, unexpectedly low incidence was reported for officially recognised occupational respiratory diseases in Turkey over 2013–2023 (19), despite epidemiological reports indicating that it should be higher. The authors suggest that low incidence in their study may reflect underestimation of clinical evidence by patients and physicians as well as Turkish restrictive regulation of at least 10 % disability to qualify as occupational illness, for which reason many cases may stay under the official radar.

Although the SGK registry does not provide diagnosis-specific data by industrial sectors, manufacturing, mining, construction, and healthcare have consistently accounted for the majority of recognised occupational diseases nationwide (7, 8). While these sectors are globally recognised for high biomechanical demands and tendon disorders (2, 5, 20,21,22), the aggregate nature of our data does not allow for direct comparison, and any association remains contextual and hypothetical. While the diagnoses are consistent with reported exposure-response relationships (17, 20, 22), the massive prevalence of upper-extremity disorders – lateral epicondylitis in particular – over spinal disorders points to a distinct institutional pattern easily recognising pathologies with highly localised, clinically clear-cut, and occupation-specific biomechanical causality, such as epicondylitis and tendonitis as opposed to multi-factorial conditions like chronic low back pain or lumbar disc herniations, often dismissed by medical boards as generic, age-related degenerative processes, leading to their systemic exclusion from official occupational statistics (22,23,24).

Furthermore, considering the international evidence that MSDs should constitute the most common category of occupational diseases (25,26,27,28), the compensation-oriented legal framework in Turkey seems to create administrative barriers that may result in underreporting (8, 11, 19, 29). The disproportionately low recognised incidence observed in our study suggests potential limitations in surveillance and recognition mechanisms rather than a genuinely low burden of disease (20, 21, 30, 31).

As we mentioned in the Methods section, regulatory limitations in recording cases that do not match the strict compensation-driven rules can arguably place such cases in the so-called Group Z (diseases outside the standard list). It is quite indicative that this category accounts for 33.0 % of all occupational cases and as many as 48.0 %of female worker cases. Under the Turkish legal framework, Group Z includes conditions that fell outside the rigid, predefined statutory diagnostic lists (Groups A to E) but achieved legal recognition because clinicians and the SGK Medical Board established a definitive, work-related causality. The idea that nearly half of all female occupational MSD cases could only clear the adjudication pipeline via this outside-the-list pathway suggests that the existing standard list system may be historically biased toward classic, male-dominated heavy physical labour exposures, thereby failing to proactively categorise modern ergonomic hazards predominantly faced by the female workforce.

The reversal of incidence rates observed in 2020 and 2021 – where female incidence equalled or exceeded male – warrants careful epidemiological interpretation. Several non-mutually exclusive mechanisms may account for this shift. First, women are disproportionately concentrated in sectors such as healthcare, services, and light manufacturing (e.g. textiles), which feature high repetitiveness and static postures conducive to upper-extremity MSDs like lateral epicondylitis and tenosynovitis (3, 5). Turkish local studies have already demonstrated a high baseline prevalence of musculoskeletal symptoms among surgical nurses and textile workers within these female-dominated cohorts (9, 10). During the COVID-19 pandemic, these specific sectors operated under unprecedented workloads, potentially accelerating clinical manifestation and subsequent official recognition of MSDs among female workers.

Concurrently, pandemic supply-and-demand shocks, lockdowns, and workplace restrictions disrupted production networks, reduced operational capacity, and shortened working hours in male-dominated heavy industrial settings (32,33,34). These disruptions temporarily may have suppressed workplace exposure and registry capture in high-risk male occupations, temporarily altering the male-dominated baseline typical of industrial economies (26, 27).

### Study limitations

The primary limitation of this study is that the national SGK registry completely lacks sector-specific classification codes (such as the European Community Statistical Classification of Economic Activities, i.e. NACE identifiers) for individual disease entries. Instead, it relies only on aggregated administrative data, which renders impossible for us to compare them with literature reports to verify our hypothesis that the true disease burden may be underestimated.

Furthermore, the official aggregate reporting precludes adjustment for age. However, the key limitation is that the registry includes only employees insured under the universal health insurance law (No. 5510, Article 4/1-a) (12) and ignores the self-employed and informal workers (such as unregistered, gig, or part-time workers not included in the safety net).

Yet these limitations reinforce our argument that the current registry likely underreports the national disease burden owed to MSDs, as informal sectors often feature high exposure to ergonomic hazards without systematic surveillance.

## CONCLUSION

Our findings raise the issue that there may be a substantial gap between the registry-recognised incidence and the hypothesised true burden, that is, that occupational MSDs may be grossly under-recognised and underreported by the current registry in Turkey. To bridge this gap, national policy must transition from a passive compensation-based system towards an active, prevention-oriented surveillance that incorporates proactive ergonomic risk assessments. Aligning national practices with international standards is essential to accurately capture and ultimately reduce the long-term burden of work-related MSDs. To that end, future research should rely on individual insurance records to map the burden and inform policy changes.
